# Response of Sport Horses to Different Formulations of Equine Influenza Vaccine

**DOI:** 10.3390/vaccines8030372

**Published:** 2020-07-10

**Authors:** Johanna Entenfellner, Jacinta Gahan, Marie Garvey, Cathal Walsh, Monica Venner, Ann Cullinane

**Affiliations:** 1 School of Veterinary Medicine, Bischofsholer Damm 15, 30173 Hannover, Germany; johannaentenfellner@icloud.com; 2 Irish Equine Centre, Johnstown, Naas, Co. Kildare, W91 RH93 Johnstown, Ireland; JGahan@irishequinecentre.ie (J.G.); MGarvey@irishequinecentre.ie (M.G.); 3 Department of Mathematics and Statistics, University of Limerick, V94 T9PX Limerick, Ireland; Cathal.Walsh@ul.ie; 4 Pferdeklinik Destedt GmbH, Destedt, Trift 4, 38162 Cremlingen, Germany; MVenner@gmx.de

**Keywords:** equine influenza, inactivated, subunit, recombinant vaccines, sport horse, antibody, reactogenicity

## Abstract

The international governing body of equestrian sports requires that horses be vaccinated against equine influenza within 6 months and 21 days of competing. The aim of this study was to compare the antibody response of young sport horses to six-monthly booster vaccination with equine influenza vaccines of different formulations. An inactivated vaccine was allocated to 35 horses and subunit and recombinant vaccines were allocated to 34 horses each. After vaccination, all horses were monitored for evidence of adverse reactions. Whole blood samples were collected at the time of vaccination and on nine occasions up to six months and 21 days post vaccination. Antibodies against equine influenza were measured by single radial haemolysis. Transient fever and injection site reactions were observed in several horses vaccinated with each vaccine. Only two horses failed to seroconvert post booster vaccination but there was a delayed response to the recombinant vaccine. The antibody response to the recombinant vaccine was lower than that induced by the whole-inactivated and subunit vaccines up to three months post vaccination. Thereafter, there was no significant difference. By six months post vaccination, the majority of horses in all three groups were clinically but not virologically protected. There was minimal decline in antibody titres within the 21-day grace period.

## 1. Introduction

The economic impact of equine influenza is minimised by vaccination with vaccines that decrease virus shedding and clinical signs [1,2]. However, the protection induced by vaccination is short lived and multiple doses of vaccine are required to protect highly mobile horses and safeguard equestrian events [3,4]. The vaccines that are most widely available globally are of three types: whole inactivated, subunit and recombinant [2]. The objective of this study was to compare the humoral immune response after booster vaccination with a whole-inactivated vaccine (Duvaxyn IE-T Plus), an immune-stimulating complex (ISCOM)-based subunit vaccine (EquipFT) and a canarypox recombinant vaccine (Proteq Flu-Te). Antibodies against the envelope glycoprotein haemagglutinin (HA) have been demonstrated to protect against equine influenza both in experimental challenge studies and in outbreaks in the field [5,6,7,8,9]. These antibodies can be measured by the haemagglutination inhibition (HI) test or the single radial haemolysis (SRH) test but only the SRH test is standardised internationally with reference sera available from the European Directorate for the Quality of Medicines (EDQM) [10,11]. SRH antibody titres are established correlates of protection. If the vaccine strain is closely related to the challenge virus, antibody titres ≥150 mm^2^ are associated with protection against infection commonly referred to as virological protection, and antibody titres ≥85 mm^2^ are associated with clinical protection but horses may shed virus [5,6,12]. Thus, evaluation of SRH antibody titres is accepted as an evidence-based approach to the examination of vaccine efficacy in the field where experimental virus challenge is not feasible. The majority of virus challenge studies are limited to primary vaccination consisting of two or three doses of vaccine and rarely shed any light on the status of horses that have been regularly vaccinated [2]. In the past, we have compared the response of Thoroughbred weanlings and National Hunt racehorses to primary vaccination and annual booster vaccination, respectively, with inactivated, subunit and recombinant vaccines [13,14]. In this study, we compared the response of young sport horses to six-monthly booster vaccination with similar vaccines.

## 2. Materials and Methods

### 2.1. Horses

This study was undertaken on a single farm to compare the humoral response to booster vaccination with three different EI vaccines. One hundred and three horses were available for vaccination. Horses were chosen sequentially for vaccination with the same product, with 35 in the first group and 34 in the other groups, with the order of vaccines chosen at random. Animals were followed up longitudinally with repeated samples drawn at ten time points. After approval by the Ethics Committee of the Veterinary School of Hannover, the study was registered by the authorities of the Meclenburg-Vorpommern. The horses ranged in age from 2 to 7 years old (median: 3 years) at the time of vaccination when, on average, it was 172 (±11.4) days since their last vaccination. The horses had received from 4 to 15 (mean 6.5 ± 2.2) doses of vaccine prior to this study.

### 2.2. Vaccines

A whole-inactivated vaccine Duvaxyn IE-T Plus (Elanco Animal Health, Eli Lilly Ltd., Basingstoke, United Kingdom), an ISCOM subunit vaccine EquipFT (Zoetis, Parsippany-Troy Hills, NJ, USA) and a recombinant canarypox vaccine ProteqFlu-Te (Merial S.A.S./Boehringer Ingelheim, 29 Avenue Tony Garnier, 69007, Lyon, France) were all purchased commercially for inclusion in this study. The different adjuvants and composition of each of the three vaccines are shown in Table 1.

### 2.3. Vaccination

The horses in this study were randomly allocated one of the three vaccines. The inactivated vaccine was allocated to 35 horses and the subunit and recombinant vaccines were allocated to 34 horses each. Gender was not a factor in vaccine allocation but mares, geldings and stallions were included in each group. Vaccines were administered by deep intra muscular injection which was performed by the attending veterinary surgeon. Following vaccination, all horses were observed for evidence of adverse local and/or systemic reactions. Rectal temperatures were taken daily for three days post vaccination.

### 2.4. Collection of Samples

Whole blood samples were collected by the attending veterinary surgeon on the day of vaccination (D0) and days seven (D7), 14 (D14), 21 (D21), 60 (D60), 90 (D90), 121 (D121), 150 (D150), 180 (D180) and 201 (D201) post booster vaccination. Due to animal handling limitations, some of the horses could not be sampled at one or more of the sampling times and horses were lost to the study at different times as they were sold. The number of horses sampled on each occasion is shown in Table 2.

### 2.5. Serology

Antibodies against two H3N8 viruses A/equine/Meath/1/2007 (Florida Clade 2) and A/equine/South Africa/4/2003 (Florida Clade 1) were measured using the SRH test as previously described [15]. A/equine/Meath/1/07 demonstrates 100% HA nucleotide identity to the Florida clade 2 World Organisation for Animal Health (OIE)-recommended vaccine strain A/equine /Richmond/1/07 [16]. A/equine/South Africa/4/2003 is one of the OIE-recommended Florida clade 1 vaccine strains. The degree of HA amino acid identity between the strains used in the vaccines and the strains used to conduct SRH is presented in Table 3. The area of haemolysis resulting from the lysis of equine influenza antigen-coated SRBCs by the antibody in the test sera were expressed in mm^2^. Mean H3N8 antibody values, i.e., against A/equine/Meath/1/2007 and A/equine/South Africa/4/2003, were calculated from SRH results obtained and these are presented in Supplementary Table S1. A significant increase in antibody titre was defined as an increase of ≥25 mm^2^ [9]. Similarly, a significant decline in antibody titre was defined as a decrease of ≥25 mm^2^. The laboratory investigator was blind to vaccine allocation to individual horses. The response to tetanus toxoid following vaccination was not examined during this study.

### 2.6. Statistical Analysis

All statistical analysis was carried out on the open-source package R version 3.1.1 (CRAN, Dublin, Ireland) [17]. A significance level of *p* < 0.05 was used for all statistical tests. Pearson’s correlation coefficient was used to examine whether there was a correlation between age and number of vaccines received prior to the study. A Kruskal–Wallis test was used to examine the association between baseline titre and categorical variables such as gender and vaccine type. Linear models, using main effects only, were used to examine the association between continuous variables, such as age at first vaccination and baseline titre. Pearson’s chi-squared test was used to determine if there was a significant difference between the vaccines in their association with pyrexia and local inflammation. Data were analysed using a repeated-measures analysis of variance and post hoc testing was carried out with Tukey’s HSD. Residuals were examined visually for normality. The area under the curve (AUC) as described by Heldens et al. (2002) [18] was calculated by the trapezoidal rule and used as the metric for the repeated-measures analysis of antibody levels. To examine the change in titre between the final two time points in the study, a paired *t*-test was used for each vaccine.

## 3. Results

### 3.1. Pre-Existing Immune Status

The equine population in this study was investigated for factors that influenced baseline SRH titre. In univariate models, the greater the number of vaccinations previously received and the older the horse, the greater the baseline SRH titre on D0 (*p* < 0.001). However, these factors are confounding, as the older horses had received more vaccinations (Pearson’s correlation co-efficient = 0.97). Gender (*p* > 0.9) or age at first vaccination (*p =* 0.8) did not significantly affect the baseline titre. The D0 baseline SRH titre was similar for the three vaccine groups (*p =* 0.1).

### 3.2. Reaction to Vaccination

Four horses mounted a febrile response post vaccination with the whole-inactivated vaccine. A transient increase in temperature was observed in two horses on day two (D2) (38.5 and 38.7 °C) and in two horses on day 3 (D3) (38.5 and 38.6 °C) post vaccination. Three horses developed (one on D2 and two on D3) a small local swelling at the injection site, two of which were painful on palpitation.

Five horses mounted a febrile response post vaccination with the subunit vaccine. A transient increase in temperature was observed in four horses on D2 (38.5–38.7 °C) and in one horse on D3 post vaccination (38.5 °C). Seven horses experienced transient injection site reactions three of which were painful on palpitation. Two of the horses with local reactions were pyrexic.

Seven horses mounted a febrile response post vaccination with the recombinant vaccine. An increase in temperature was observed in five horses on D2 (38.6–38.9 °C) and two horses on D3 (38.6 and 38.7). Two of the horses that spiked a temperature on D2 were still pyrexic (39.5–38.7 °C) on D3, and one of these developed an injection site reaction on D2. In total, nine horses developed injection site reactions, two of which were painful on palpitation.

There was no significant difference between the vaccine groups in the incidence of pyrexia (*p* = 0.6) or local inflammation (*p* = 0.1).

### 3.3. Immune Response to Booster Vaccination

**Figure 1:**
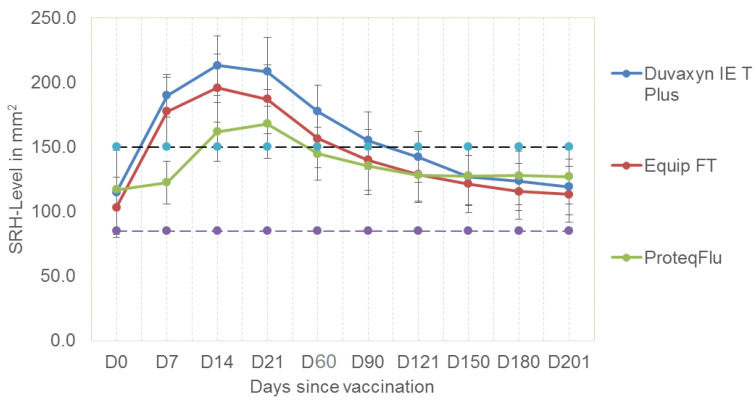
The mean SRH values with standard deviation bars for the three vaccine groups, those vaccinated with a whole-inactivated vaccine (Duvaxyn IE-T Plus), an ISCOM subunit vaccine (EquipFT) and a recombinant canarypox vaccine (ProteqFlu-Te), at each sampling point, i.e., the day of vaccination (D0) and days 7, 14, 21, 60, 90, 121, 150, 180 and 201 post vaccination. The black dashed line with blue circles indicates 150 mm^2^, the threshold level of SRH antibodies associated with virological protection. The purple dashed line indicates 85 mm^2^, the threshold level of SRH antibodies associated with clinical protection.

The mean SRH values for the three vaccine groups at each sampling point are illustrated in Figure 1 and all the results for individual horses are presented in Supplementary Table S1. All of 33 horses vaccinated with the subunit vaccine and all but one of the horses vaccinated with the whole-inactivated vaccine seroconverted by D7. In contrast, none of the horses vaccinated with the canarypox vaccine seroconverted by D7. Twenty two of 31 horses vaccinated with the canarypox vaccine seroconverted between D7 and D14, and two of the remaining horses seroconverted between D14 and D21.

This study did not include experimental challenge but based on antibody titre on D21, all horses vaccinated with the whole-inactivated vaccine tested were expected to be virologically protected, as were 97% of those vaccinated with the ISCOM subunit vaccine but only 73% of the horses vaccinated with the canarypox recombinant vaccine. After D21, the SRH antibody titres decreased for all vaccine groups. By D60, although 61% of horses vaccinated with the whole-inactivated vaccine had experienced a significant decline (≥25 mm^2^), 93% of the horses sampled were virologically protected with titres ≥150 mm^2^. This compared favourably with 62% and 48% of the horses vaccinated with the subunit and canarypox vaccines, respectively. Titres continued to decline for all vaccine groups and by three months post vaccination (D90), only 50% of the horses vaccinated with the inactivated vaccine were expected to be virologically protected, as were 27% and 30% of those vaccinated with the subunit and canarypox vaccines, respectively. By six months post vaccination, with very few exceptions, the horses in all three groups were expected to be clinically but not virologically protected.

Tukey’s HSD was carried out to examine significant differences in performance between the three vaccines included in this study. Results of this analysis are shown in Table 4 and include the comparison of all possible pairs of means, that is they apply simultaneously to the set of all pairwise comparisons. The antibody response of the horses vaccinated with the whole-inactivated and subunit vaccines was not significantly different at any time point but the response of both groups was significantly better than that elicited by vaccination with the canarypox recombinant vaccine up to 90 days post vaccination. At 121, 150, 180 and 201 days post vaccination, there was no significant difference between the antibody response of the three groups. 

The area under the curve (AUC) against the SRH antigens tested was also calculated for each of the three vaccines. This was performed using complete case data (*n* = 52) up to day 180, since missing data resulted in only 42 complete cases at day 201. The magnitude of the immune response measured by AUC analysis revealed a significantly lower antibody response for the subunit and canarypox vaccines compared to the whole-inactivated vaccine. There was a significant difference between the AUC for the subunit and whole-inactivated vaccine (*p =* 0.046), and the canarypox recombinant vaccine and whole-inactivated vaccine (*p =* 0.008) but not between the subunit and canarypox recombinant vaccine (*p =* 0.67).

A question of interest is the change in titre during the FEI grace period, from day 180 to day 201. For each vaccine, this change was examined using a paired *t*-test. For Duvaxyn IE-T Plus, there was a decline of 3.79 mm^2^ (95% CI 2.0 to 5.6); for EquipFT, there was a a decline of 2.8 mm^2^ (95% CI 0.6 to 5.0); and for ProteqFlu TE, there was no statistically significant change—a decline of 0.02 mm^2^ (95% CI 2.8 to −2.8).

## 4. Discussion

This study compared the antibody responses to commercial equine influenza vaccines for six months following a biannual booster. A subset of the serology results were previously included in a global project to establish within the context of existing OIE standards, a science-based rationale to identify the ideal time period for equine influenza vaccination prior to shipment [19]. The current study focused on a comparison of vaccine performance including adverse effects in a population consisting of young regularly vaccinated sport horses. At the time of vaccination, their mean SRH antibody titre of 112 mm^2^ was slightly lower than that of 123 mm^2^ recorded previously for horses of a similar age in Irish racing yards [15] but is considered to afford clinical protection from field viruses closely related to the virus in the vaccine [20]. The horses in the current study had commenced their primary course of two doses of vaccine one month apart from seven to 12 months of age and thereafter had received six-monthly boosters. The manufacturers’ of Proteq Flu-TE and Duvaxyn IE-T Plus recommend annual boosters and the manufacturer of Equip recommends 12 to 15 month boosters after a primary course of three vaccinations. However, the practice of vaccinating horses, particularly sport horses, at six-monthly intervals is not uncommon. In the early 1980s, the Federation Equestre Internationale (FEI), the international governing body of equestrian sports, introduced a mandatory primary vaccination schedule of three doses of vaccine followed by annual revaccination. However, in 2004, the FEI approved a rule change requiring all horses competing in FEI competitions to be vaccinated within 6 months and 21 days of competing. This was in response to several reports that more frequent vaccination significantly reduced the risk of influenza outbreaks [21,22,23]. Vaccination breakdown has been recorded as occurring primarily, but not exclusively, among young horses which had not received a booster vaccination within the previous six months [4].

In this study, transient fever and injection site reactions were observed in several horses vaccinated with each vaccine. Such adverse reactions are documented by the vaccine manufacturers and, although one manufacturer suggests that these reactions are rare, no significant difference was found between the three vaccines in this study. A prominent acute phase response to vaccination with equine influenza vaccines has been documented [24]. Horses respond not only to the viral component of a vaccine but also to non-target antigens such as proteins derived from the embryonated eggs or cells in which the virus is propagated, and vaccine excipients. This response varies with the individual and may increase with repeat vaccination [25]. For human athletes, it is recommended that vaccinations should be scheduled in a way that possible side effects are least likely to occur in periods of competition [26]. The FEI allow a 21-day grace period to allow for strategic vaccination scheduling. This should not jeopardise the immune status, as results of this study provide evidence that the decline in antibody titre induced by three different vaccine types within the grace period is minimal.

The horses responded well to booster vaccination with the vaccines included in the study. In a previous study, eighteen of 44 National Hunt race horses (41%) did not experience a significant increase in titre after receiving their annual booster [14] but only two of the sport horses in this study failed to demonstrate an increase of ≥25 mm^2^ post booster vaccination. The delayed response to the recombinant vaccine compared to the whole-inactivated and subunit vaccines in the current study corroborates the findings in racehorses and provides further support for the recommendation that Proteq Flu-TE be administered no later than two weeks prior to an event [27]. FEI rules permit administration of an equine influenza vaccine up to seven days before arrival at the event, which may not be adequate to provide the best protection afforded by certain vaccines.

The pattern of humoral antibody response was similar for the three vaccines and to that observed previously in racehorses [14]. The antibodies peaked between two and three weeks post vaccination and decreased sharply by three months post vaccination. In the previous study in racehorses, there was no significant difference observed between antibody response induced by any of six commercial vaccines including ProteqFlu-TE, Equip FT and Duvaxyn IE-T-Plus. However, in the current study, Tukey’s HSD indicated that on the basis of antibody titres, Equip FT and Duvaxyn IE-T-Plus were superior to ProteqFlu-TE up to three months post vaccination. Thereafter, there was no significant difference and the antibody titre induced by the three vaccines declined gradually to levels associated with clinical protection rather than virological protection.

The three vaccines used in this study represent a diversity of technologies and have proved to be efficacious in the reduction in clinical signs and virus shedding in experimental challenge studies [6,7,28,29,30,31,32]. All three have been shown to elicit both a humoral and cell mediated response [13,14,30,31,32]. Many factors can affect the efficacy of an equine influenza vaccine including the relatedness of the virus strains in the vaccine to circulating virus. It has been demonstrated in the field that horses require higher antibody titres for protection if there is a mismatch between the circulating virus and the vaccine strains [8]. The recombinant vaccine ProteqFlu-TE did not induce the same level of humoral antibody response as Duvaxyn IE-T Plus and Equip TE, but was the only vaccine in this study to be updated in line with the OIE recommendations for equine influenza vaccine strain composition [33], suggesting that its protection threshold may be lower than vaccines with older strains. Furthermore, it is important to consider that this is a second-generation live recombinant vaccine which presents the influenza HA to the host in a manner that is considered to mimic natural infection and stimulate a complex immune response. However, the measurement of cellular or mucosal immunity was beyond the scope of this study and the most reliable evaluation of vaccine efficacy can only take place in the field when regularly vaccinated horses are exposed to virus during an outbreak of influenza.

## 5. Conclusions

Transient fever and injection site reactions were observed in several horses vaccinated with the subunit, whole-inactivated and recombinant vaccines but there was no significant difference in reactogenicity to the different formulations. All three vaccines elicited a significant antibody response but AUC analysis revealed a significantly lower response for the subunit and canarypox vaccines compared to the whole inactivated vaccine. There was a delayed response to the recombinant vaccine and for up to three months post vaccination the antibody titres were lower than those elicited in response to vaccination with the whole-inactivated and subunit vaccines. From three to six months post vaccination there was no significant difference in vaccine performance.

## Figures and Tables

**Table 1:** Vaccine product details.

Vaccine Producer	Nature	Adjuvant	Virus Strains	Tetanus Toxoid
Duvaxyn IE-T Plus (Elanco)^®^	Inactivated whole virus	Carbomer Aluminium hydroxide	A/eq/Prague/56 (H7N7) A/eq/Suffolk/89 (H3N8) A/eq/Newmarket/1/93 (H3N8)	>150 I.U.
Equip FT (Zoetis)^®^	Subunit	ISCOM Quillaic acid derivative Aluminium-phosphate	A/eq/Newmarket/77 (H7N7) A/eq/Kentucky/98 (H3N8) A/eq/Borlange/91 (H3N8)	100 Lf
ProteqFlu TE (Merial)^®^	Canarypox-recombinant	Carbomer	A/eq/Ohio/03 (H3N8) A/eq/Richmond/1/07 (H3N8)	>30 I.U.

ISCOM = immune-stimulating complexes, IU = international unit, and Lf = limes flocculation units.

**Table 2:** Number of horses sampled at each time point post vaccination.

Vaccine	D0	D7	D14	D21	D60	D90	D121	D150	D180	D201
Duvaxyn IET Plus	35	34	32	34	29	30	30	23	23	20
Equip FT^®^	34	33	34	33	26	26	26	20	20	15
Proteq Flu Te^®^	34	34	31	30	23	23	23	18	18	14
Total	103	101	97	97	78	79	79	61	61	49

**Table 3:** The degree of HA amino acid identity (%) between the strains used in the vaccines and the strains used to conduct SRH, A/equine/Meath/1/2007 and A/equine/South Africa/4/2003.

Vaccine	Nature	Vaccine Virus Strains	Amino Acid Identity (%)	
			A/eq/South Africa/4/03	A/eq/Meath/1/07
Duvaxyn IE-T Plus (Elanco)^®^	Inactivated whole virus	A/eq/Prague/56 (H7N7) A/eq/Suffolk/89 (H3N8) A/eq/Newmarket/1/93 (H3N8)	47.69 97.27 96.96 *	47.51 96.90 96.96 *
Equip FT (Zoetis)^®^	Subunit	A/eq/Newmarket/77(H7N7) A/eq/Kentucky/98 (H3N8) A/eq/Borlange/91 (H3N8)	47.51 96.04 * 92.70 *	47.34 96.04 * 92.40 *
ProteqFlu TE (Merial)^®^	Canarypox-recombinant	A/eq/Ohio/03 (H3N8) A/eq/Richmond/1/07 (H3N8)	100 98.90	98.90 100

* % identity to Haemagglutinin 1 only.

**Table 4:** Post hoc Tukey’s HSD comparisons. Performance of the vaccines at different time points post booster vaccination compared using Tukey’s HSD. Negative values indicate a greater change from baseline for the latter vaccine in each paired comparison.

Time Points	95% Confidence Interval		*p* Value
	Lower	Upper	
**D0–D7**			
EquipFT-Duvaxyn IE-T Plus	−11.61	14.22	>0.9
ProteqFLU TE-Duvaxyn IE-T Plus	−81.04	−55.4	<0.001 *
ProteqFLU TE-EquipFT	−82.44	−56.61	<0.001 *
**D0–D14**			
EquipFT-Duvaxyn IE-T Plus	−25.42	12.9	0.7
ProteqFLU TE-Duvaxyn IE-T Plus	−72.83	−33.6	<0.001 *
ProteqFLU TE-EquipFT	−66.3	−27.7	<0.001 *
**D0–D21**			
EquipFT-Duvaxyn IE-T Plus	−27.1	7.50	0.4
ProteqFLU TE-Duvaxyn IE-T Plus	−62.2	−26.75	<0.001 *
ProteqFLU TE-EquipFT	−52.7	−16.71	<0.001 *
**D0–D60**			
EquipFT-Duvaxyn IE-T Plus	−24.73	11.16	0.7
ProteqFLU TE-Duvaxyn IE-T Plus	−54.48	−17.38	<0.001 *
ProteqFLU TE-EquipFT	−48.17	−4.18	<0.01 *
**D0–D90**			
EquipFT-Duvaxyn IE-T Plus	−18.59	14.72	>0.9
ProteqFLU TE-Duvaxyn IE-T Plus	−41.14	−6.69	<0.01 *
ProteqFLU TE-EquipFT	−48.17	−4.18	<0.01 *
**D0–D121**			
EquipFT-Duvaxyn IE-T Plus	−15.55	14.69	>0.9
ProteqFLU TE-Duvaxyn IE-T Plus	−34.14	−2.86	0.016 *
ProteqFLU TE-EquipFT	−34.22	−1.9	0.02 *
**D0–D150**			
EquipFT-Duvaxyn IE-T Plus	−4.98	24.16	0.3
ProteqFLU TE-Duvaxyn IE-T Plus	−18.04	12.27	0.9
ProteqFLU TE-EquipFT	−28.26	3.05	0.1
**D0–D180**			
EquipFT-Duvaxyn IE-T Plus	−7.3	21.71	0.5
ProteqFLU TE-Duvaxyn IE-T Plus	−14.03	15.84	>0.9
ProteqFLU TE-EquipFT	−21.71	9.13	0.6
**D0–D201**			
EquipFT-Duvaxyn IE-T Plus	−7.95	25.05	0.4
ProteqFLU TE-Duvaxyn IE-T Plus	−14.2	19.5	>0.9
ProteqFLU TE-EquipFT	−23.9	12.0	0.7

* significant difference.

## Supplementary Materials

The following are available online at https://www.mdpi.com/2076-393X/8/3/372/s1, Table S1: SRH antibody titres (area of haemolysis, mm^2^) against A/equine/Meath/1/2007 and A/equine/South Africa/4/2003, and mean H3N8 antibody titres calculated for individual horses.
